# Supplementary figures and images for: Pro-atrial natriuretic peptide and pro-adrenomedullin before cardiac surgery in children. Can we predict the future?
Source: PLoS One. 2020 Jul 23;15(7):e0236377. doi: 10.1371/journal.pone.0236377 (PMC7377469; doi:10.1371/journal.pone.0236377)

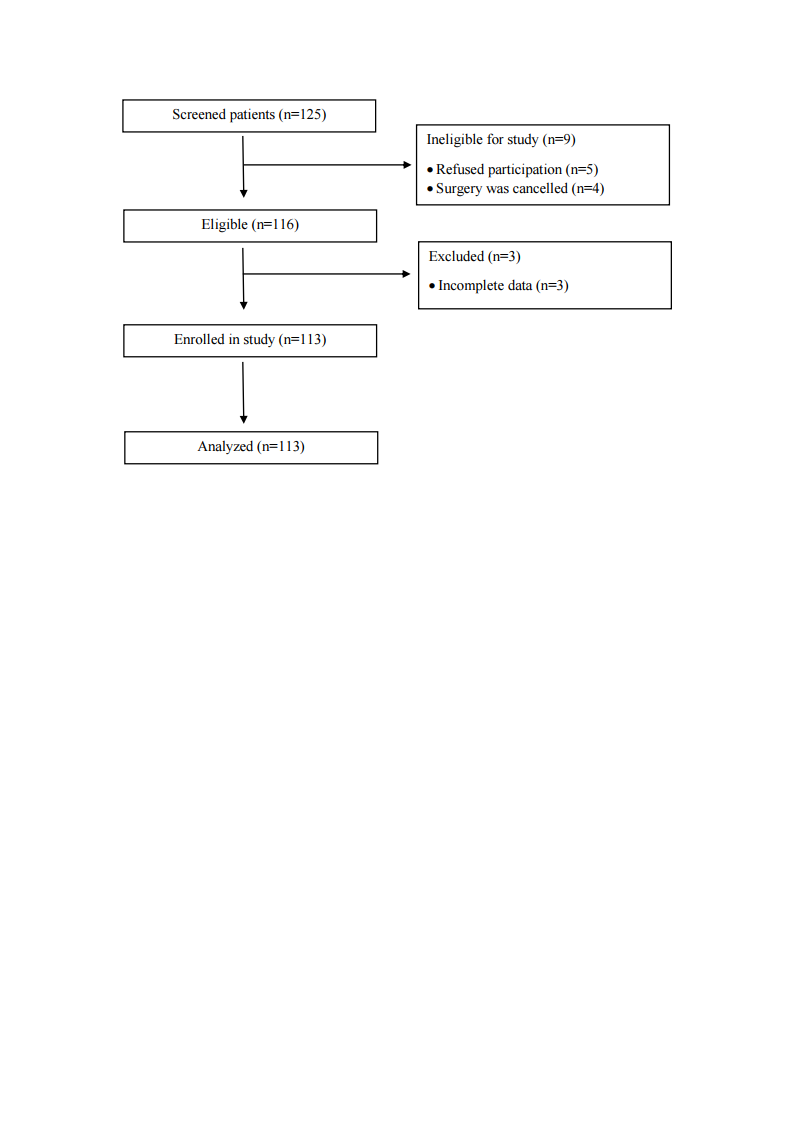

Supplement: S1 Fig — (TIFF) [file pone.0236377.s001.tiff]
